# Assessment of Brain Function in Patients With Cognitive Impairment Based on fNIRS and Gait Analysis

**DOI:** 10.3389/fnagi.2022.799732

**Published:** 2022-05-24

**Authors:** Zehua Wang, Ke Ren, Deyu Li, Zeping Lv, Xiang Li, Xiaoli He, Daifa Wang, Wenyu Jiang

**Affiliations:** ^1^Key Laboratory of Biomechanics and Mechanobiology, Ministry of Education, Beijing Advanced Innovation Center for Biomedical Engineering, School of Biological Science and Medical Engineering, Beihang University, Beijing, China; ^2^National Research Center for Rehabilitation Technical Aids, Beijing, China; ^3^Shenzhen Institutes of Advanced Technology, Chinese Academy of Sciences (CAS), Shenzhen, China; ^4^Ningxia University, Yinchuan, China; ^5^Jiangbin Hospital of Guangxi Zhuang Autonomous Region, Nanning, China

**Keywords:** cognitive impairment, fNIRS, brain functional connectivity, dual-task walking, gait analysis

## Abstract

**Background:**

Early detection of mild cognitive impairment is crucial in the prevention of Alzheimer’s disease (AD). This study aims to explore the changes in gait and brain co-functional connectivity between cognitively healthy and cognitively impaired groups under dual-task walking through the functional near-infrared spectroscopy (fNIRS) and gait analysis devices.

**Method:**

This study used fNIRS device and gait analysis devices to collect the data of 54 older adults. According to the Mini-mental State Examination (MMSE) and the Montreal Cognitive Assessment (MoCA) scales, the older adults were cognitively healthy (control group) and cognitively impaired (experimental group), of which 38 were in the control group and 16 were in the experimental group. The experiment was divided into a total of three sets of task experiments: a walking-only experiment, a dual-task walking-easy (DTW-easy) experiment, and a dual-task walking-difficult (DTW-difficult) experiment.

**Main Result:**

For the cognitively impaired and cognitively healthy populations, there were no significant differences in overall functional connectivity, region of interest (ROI) connection strength, and gait performance during single-task walking between the two groups.Whereas the performances of DTW differed significantly from the single-task walking in terms of between-group variability of functional connectivity strength change values, and ROI connection strength change values in relation to the dual-task cost of gait. Finally, the cognitively impaired group was significantly more affected by DTW-difficult tasks than the cognitively healthy group.

**Conclusion:**

This study provides a new approach to assist in the diagnosis of people with cognitive impairment and provides a new research pathway for the identification of cognitive impairment.

## Introduction

Alzheimer’s disease (AD) is a neurodegenerative disorder whose main clinical manifestation is cognitive impairment (Goedert and Spillantini, 2006). Cognitive impairment generally follows neurodegenerative disorders and can also be used as a precursor to neurodegenerative disease to implement interventions to prevent neurodegenerative disorder (Joe and Ringman, 2019). Although the main clinical manifestation of cognitive impairment is impairment in learning and memory thinking, motor impairment or gait impairment is also an important dimension in the early screening of neurodegenerative disorder in older adults (Li et al., 2016; Bragatto et al., 2017).

Gait impairment is common in older adults with cognitive impairment, and motor impairment usually occurs early in cognitive decline. Compared with the controlled group, those with cognitive impairment have poor performance in motor accuracy, motor complexity, balance, and limb coordination (Hausdorff and Buchman, 2013). In addition, Montero-Odasso conducted a comparative study between groups with cognitive impairment and normal individuals. It was also found that the gait performance of the cognitively impaired group has poorer gait performance than the cognitively healthy group (Montero-Odasso et al., 2014). It has also been hypothesized that vascular or neurodegenerative mechanisms, or both, may contribute to the simultaneous decline of movement and cognition. However, cognitive and motor dysfunction do not show causality in the cognitively impairment group, but rather reflect that both cognitive and motor control circuits were responsible for the brain networks. In contrast, adding markers of motor function, such as dual-task gait, to the biomarkers of mild cognitive impairment may help to better identify cognitive sub-types and predict the transition from mild cognitive impairment to Alzheimer’s disease and other dementia (Verghese et al., 2008).

Gait is usually considered s an automatic human behavior, but studies have shown that cognitive ability plays a decisive role in gait initiation, stopping, turning, and rhythmic shifts. In particular, the dependence of gait on cognitive ability is more obvious in older adults with cognitive impairment (Gillain et al., 2013). Dual-task walking (DTW) consists of a primary task and a sub-task, in which walking is the primary task and the other task is the sub-task (Falbo et al., 2016; Bragatto et al., 2017). During dual-task walking, gait will be affected compared to single-task walking. Strike and Taylor (2009) investigated the direct parameters of gait of subjects when performing turns compared to straight walking. The results showed that there was a significant reduction in gait during turns compared to straight walking in terms of speed, step length, and step frequency (Strike and Taylor, 2009). Direct gait data are behavioral data that are directly accessible through gait experiments. Indirect gait data are parameters obtained by secondary integration calculations of direct data. In the study of coefficient of variation (COV) of gait, the gait variability under each test condition during step training was quantified by calculating the step parameters, such as the speed variability, step time variability, step width variability, and support time variability (Springer et al., 2006; Gouelle et al., 2016; Bohrer et al., 2018).

For the neuroimaging studies of cognitively impaired groups, the commonly used traditional imaging tools mainly include electroencephalograph (EEG), functional magnetic resonance imaging (fMRI), etc. The above traditional technical tools mostly focus on collecting the resting-state or the task state in the sitting position (Machulda et al., 2003; Poil et al., 2013; Bu et al., 2020). Because of this, traditional imaging techniques, such as fMRI and EEG, require subjects to remain strictly stationary in the laboratory to reduce noise interference. In contrast, DTW studies involving cognitive impairment are generally related to cognitive ability, and the relevant experimental paradigm usually requires subjects to walk within a certain range. In addition, people with cognitive impairment are a special group of people who are emotionally and psychologically unstable. Being in an unnatural environment will make their emotions fluctuate, while the claustrophobic environment of fMRI and PET can cause their emotions to fluctuate and thus affect the accuracy of experimental results. In recent years, functional near-infrared spectroscopy (fNIRS), a brain imaging technique, has been increasingly used in various DTW studies. fNIRS utilizes the principle that oxygen and hemoglobin (HbO) and reduced hemoglobin (HbR) in cortical blood differ in their absorption of light in the near-infrared band, allowing differences in hemoglobin changes in cortical blood flow to be detected (León-Carrión and León-Domínguez, 2012; Halim et al., 2016; Wang et al., 2020). Compared with traditional imaging techniques, fNIRS is insensitive to motion artifacts while ensuring spatial and temporal resolution. Therefore, fNIRS has a higher signal-to-noise ratio, a wider tolerance range for the subject’s motion, and will not introduce excessive artifacts due to walking. In addition, fNIRS is more suitable for studies related to dual-task walking in cognitive impairment.

In this study, a dual-task walking experimental environment was constructed using fNIRS and gait acquisition equipment to investigate changes in gait parameters and brain functional connectivity strength during task performance in cognitively impaired and normal older adults. This study aims to explore the changes in gait and brain co-functional connectivity between cognitively healthy and cognitively impaired groups.

Thirty-eight cognitively healthy older adults and 16 cognitive impaired older adults were evaluated by single task and dual-task walking.

## Materials and Methods

### Subject

A total of 54 older adults aged 60 years or older were recruited for this study by recruiting subjects to the community: (a) normal vision or normal vision after correction; (b) no limb movement disorder; (c) no neurological disease to exert influence on the experiment; (d) no brain structure abnormalities caused by brain tumors and/or external injuries; (e) no obstacles to walking. All subjects signed the informed consent form and the subjects were given an experimental allowance at the end of the experiment. The cognitive abilities of the subjects were screened by the Mini-mental State Examination (MMSE) and the Montreal Cognitive Assessment (MoCA) scales. The results were determined by the scale scores. The screening process was performed by the cognitive rehabilitation physicians of Guangxi Jiangbin Hospital. This study was conducted at Guiya Health Service Center in Nanning, Guangxi.

In this study, if the MMSE score was less than or equal to 26 and the MoCA scale score was less than or equal to 23, the subjects were assigned to the cognitive impairment group (experimental group, EG) with clinical manifestations of cognitive impairment, and if the MMSE score was higher than 26 or the MoCA score was higher than 23, the subjects were assigned to the cognitively healthy group (control group, CG) without clinical manifestations of cognitive impairment. After screening, the cognitive impairment group consisted of 16 subjects and the normal group consisted of 38 subjects. The characteristics of subjects are shown in Table 1.

**Table 1:** Participants’ data.

	**CG**	**EG**
Gender (male/female)	11/27	4/12
Age (years)	67.92 ± 7.44	69.68 ± 6.52
Height (centimeter)	157.86 ± 4.63	154.68 ± 5.15
Weight (kilogram)	61.50 ± 10.83	59.06 ± 6.62
MMSE	28.36 ± 1.58	22.93 ± 2.13
MoCA	25.72 ± 2.31	17.63 ± 1.96

#### Devices

**Figure 1:**
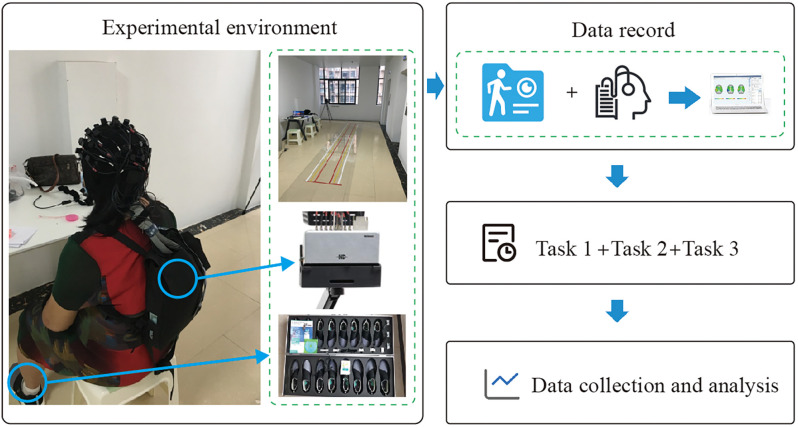
Experimental environment and procedure.

Walkway: A walkway with a length of 5 m and a width of 0.9 m is laid on the ground. A thin layer of silicone mat is laid on the bottom of the walkway to ensure non-slip portable. The whole trail is a straight line, and the starting point and ending point are set as point A and point B respectively, as shown in Figure 1.

Gait acquisition device: The gait acquisition device uses the Gibeon gait detection system produced by Dalian Qianhuan Technology to record. The system consists of three parts: gait shoes (containing pressure sensors and inertial sensors), analysis module, communication module, and the host computer. The gait shoe is a wearable device with a pressure sensor and a six-axis inertial posture sensor on the bottom of the shoe. The pressure sensor senses and records the pressure change information, so as to obtain the parameters such as the gait cycle, step time, step length, step frequency, and so on. The inertial sensor is used to analyze the step amplitude and other parameters. The communication module uses Bluetooth 2.0 Class 1 technology for data transmission, which is the hub to realize the communication between the gait shoe and the host computer. During the experiment, the analysis module first receives the signals of the pressure sensor and inertial sensor on the gait shoes by using the Velcro strapped to the relevant parts of the subject’s body, and then parses the signals and subsequently transmits them to the receiving module plugged into the computer *via* Bluetooth communication. The Bluetooth module sends the information to the software of the host computer. After the experimental acquisition, the gait parameter information is generated in the software.

fNIRS device: A portable 43-channel near-infrared functional brain imaging device (Nirsmart) from Danyang Huichuang Medical Equipment Co. was used in this study. The device consists of a head cap, optical fiber, signal collector, transmission module, host computer, etc. The head cap is used to carry the transmitter and receiver of the optical fiber. The optical fiber is used to transmit the collected near-infrared light signal, which is transmitted to the signal collector. The portable fNIRS signal collector is different from the desktop fNIRS signal collector. The portable fNIRS signal collector has a small size and wireless transmission, so it is very suitable for experiments requiring the subject to walk. Subjects can walk around freely while collecting signals. The data from the signal collector is sent to the computer through the transmission module, and the software interface of the computer displays the change of the optical signal of each channel in real-time. After the acquisition, the data file is generated and saved.

**Figure 2:**
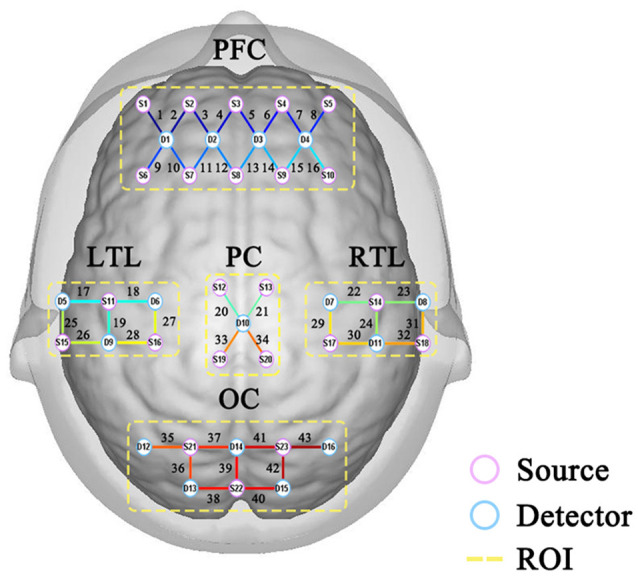
Distribution of channels.

By collating previous studies (Montero-Odasso et al., 2009; Boripuntakul et al., 2014; Zhao et al., 2021), it can be seen that most fNIRS studies focus on the prefrontal lobe. The main characteristic indexes discussed are the changes and curves of HbO and HbR in the prefrontal lobe. The activation level of the prefrontal lobe during DTW is simply explored, and the study scope is relatively limited. Therefore, 43-channel portable fNIRS covering the whole brain was used for data acquisition. After several pre-processing steps of optical density conversion, artifact identification and correction, band-pass filtering, and blood oxygen conversion, a more pure blood oxygen value is obtained. According to the requirements of the internationally-used 10/20 electrode distribution system, as shown in Figure 2, channels 1–16 of the fNIRS cover the prefrontal region (PFC) of the cerebral cortex, channels 17, 18, 19, 25, 26, 27, and 28 covers the left temporal lobe (LTL), channels 22, 23, 24, 29, 30, 31 and 32 covers the right temporal lobe (RTL), channels 20, 21, 33 and 34 cover the parietal cortex (PC), and channels 35–43 cover the occipital cortex(OC).

#### Experimental Procedure

**Figure 3:**
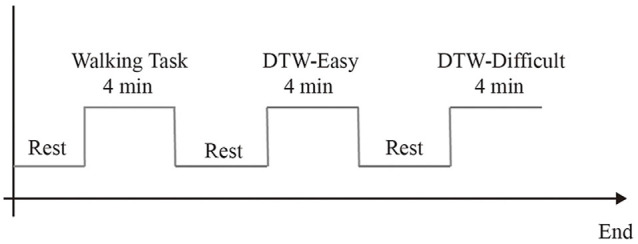
Experimental procedure.

This study was a dual-task walking experiment, which was conducted in a corridor of Guiya Community Health Service Center in Qingxiu District, Nanning, Guangxi. Before the experiment began, the staff explained the experimental procedure to the subjects and led the subjects to stand at the starting point (point A) of the pre-paved walkway. After the subjects stood at point A and facing toward point B, the staff begin to tell the subjects about the experimental content, each task is performed only once. Subjects took a 3-min break between each task. The experiment was divided into three tasks, of which task 1 was a 3-min turn-back walk between point A and point B. Task 2 was a simple dual-task experiment. Subjects walked between points A and B for 3 min while reading out numbers in the sequence (starting with 1, 2, 3) without the aid of other equipment. Task 3 was a difficult dual-task experiment. The subjects walked between point A and point B for 3 min and performed subtraction continuously (starting from 1000 to 7). Calculations were started synchronously when the subject started walking. Before each experiment, there was a 1-min baseline state data collection, during which the subjects stood steadily and looked forward, as shown in Figure 3. As the experiment consisted mainly of a walking state and a standing state, the subjects remained motionless during the standing period, kept their emotions as calm as possible, looked towards point B at the other end of the walkway, and waited for the staff to give the command to start walking. During the walking period, the subjects walked on a 5-m-long sidewalk paved in advance between point A and point B. The subjects started walking from one section, turned back and continued walking back after reaching the other end, and made a round-trip walk within 3 min. The subjects fully understand the experiment contents before the formal experiment to ensure that the formal experiment is carried out smoothly. The dual-task costing formula is as follows:

DTW_Cost = DTW gait-Single gait/Single gait × 100%.

where DTW gait denotes the gait parameters during dual-task walking and Single gait denotes the gait parameters during single-task walking.

#### Statistical Analysis

The Shapiro-Wilk test was performed to determine the conformity to the normal distribution of paired data. Considering the normality of the data, an independent sample *t*-test was chosen for the data analysis. When normality was obeyed, the independent sample *t*-test was used, otherwise, a non-parametric test was considered. It was verified that the data in this study obeyed normality overall. Very few data did not obey, but they all occurred in the 38 cases in the control group. The amount of data in the control group had reached a certain size (*n* > 30, to ensure that the overall approximation follows a normal distribution.), so the independent sample *t*-test was chosen to be used. Instead, the Mann-Whitney u test from the nonparametric test was used for the individual data in the experimental group that did not satisfy normality. In view of the fact that the raw data for dual-task costs were gait variables, and the overall data of gait variables obeyed normality, the independent sample *t*-test was used for dual-task costs. Bivariate tests were used to analyze the correlation between the amount of change in ROI and the gait dual-task cost, with Pearson coefficients and two-sided tests selected. After adjustment, *P* < 0.05 was considered statistically significant. The platform used was SPSS 22.

### Results

#### The Analysis of ROI

Regions of interest (ROI) refers to the regional division of the entire cerebral cortex according to its functionally relevant regions. In turn, the relationship between the different regions is explored through the connections between them. This study proposes to divide the cerebral cortex covered by 43 channels into seven regions.

**Figure 4:**
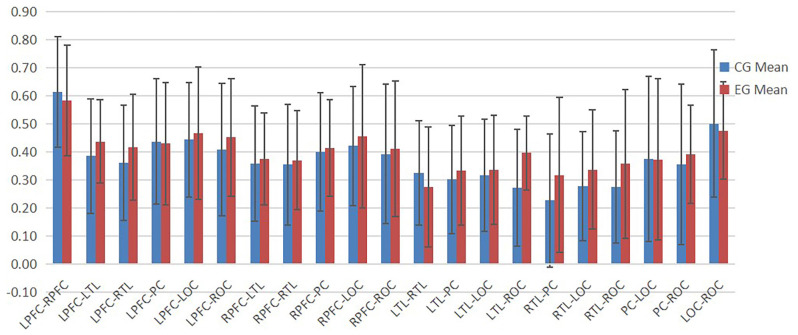
The ROI connection strength for control and cognitive impairment groups of Task 1.

**Figure 5:**
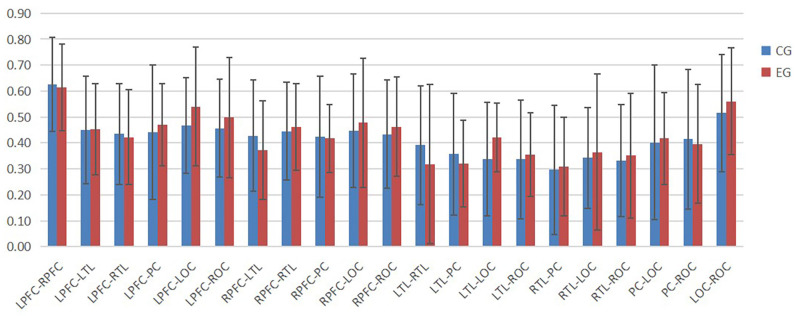
The ROI connection strength for control and cognitive impairment groups of Task 2.

**Figure 6:**
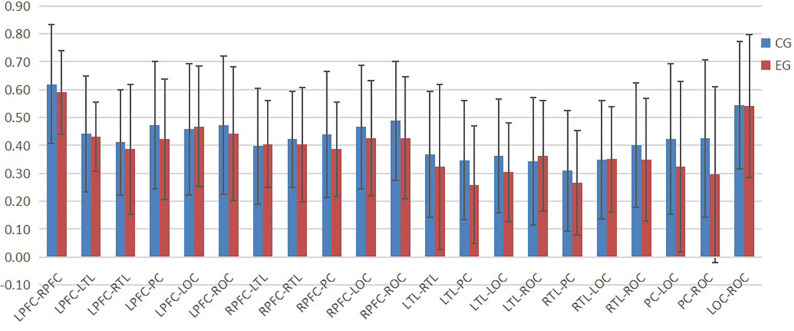
The ROI connection strength for control and cognitive impairment groups of Task 3.

According to Figures 4–6, the regional connection strength between the cognitively impaired and normal groups is different when compared in Task 1 and Task 2, whereas the regional connection strength in the normal group was generally stronger during Task 3.

#### Change in ROI Connection Strength of Task 2 and Task 3 Relative to Task 1

**Figure 7:**
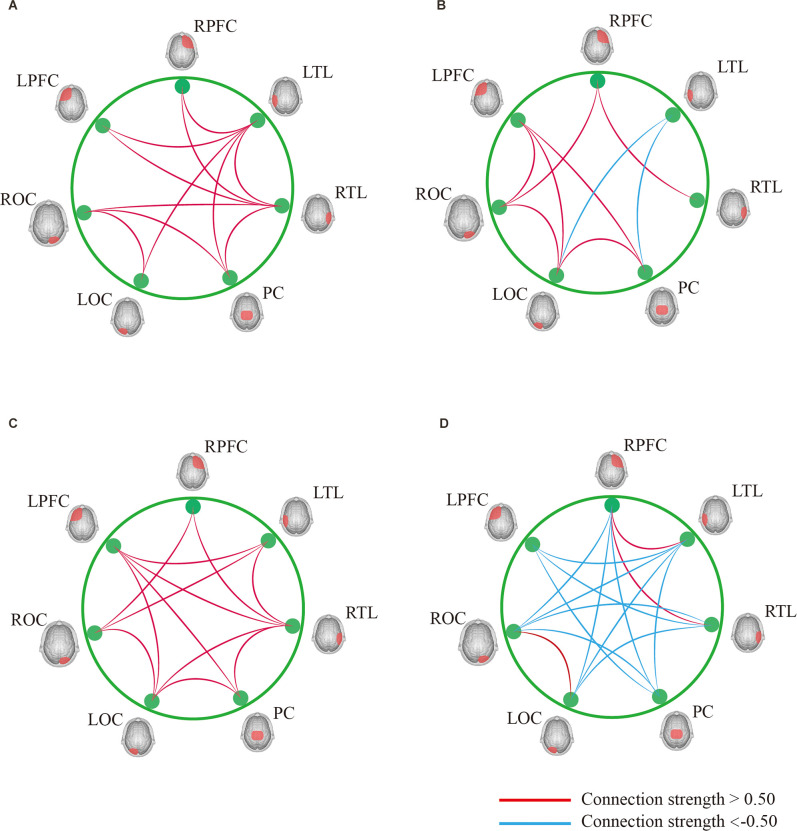
**(A)** Result of DTW-Easy with Task 1 control group. **(B)** Result of DTW-Easy with Task 1 cognitive impairment group. **(C)** Result of DTW-Difficult with Task 1 control group. **(D)** Result of DTW-Difficult with Task 1 cognitive impairment group.

In Task 2, as shown in Figure 7, the contact strength between all regions of the cognitive impairment group and the normal group increased, and there was no significant difference between the two groups. There was a greater difference in each region between the two groups, which generally increased in the normal group and decreased in the cognitive impairment group. In Task 3, there was a significant difference in the connection strength increment between RTL-PC and RTL-ROC (*t* = 2.202, *p* = 0.032 and *t* = 2.025, *p* = 0.048).

#### Analysis of the Results of Direct Gait Parameters

The statistics of the gait variables during task 1, task 2, and task 3 are in Table 2.

**Table 2:** The statistics of the gait variables during tasks 1, 2, 3.

	**Task 1**			**Task 2**			**Task 3**		
	**CG**	**EG**	**Significance**	**CG**	**EG**	**Significance**	**CG**	**EG**	**Significance**
	**Mean ± SD**	**Mean ± SD**	***p***	**Mean ± SD**	**Mean ± SD**	***p***	**Mean ± SD**	**Mean ± SD**	***p***
Step length (m)	1.02 ± 0.14*	0.90 ± 0.13*	0.006*	1.00 ± 0.15*	0.90 ± 0.09*	0.017*	0.97 ± 0.15**	0.83 ± 0.14**	0.001**
Step speed (m/s)	0.77 ± 0.16	0.72 ± 0.13	0.238	0.76 ± 0.14	0.68 ± 0.11	0.051	0.65 ± 0.15*	0.53 ± 0.14*	0.012*
Step frequency (steps/min)	91.31 ± 8.99	95.48 ± 7.91	0.114	90.51 ± 10.24	89.52 ± 11.24	0.755	80.17 ± 11.37	76.63 ± 15.11	0.349
Step time (s)	1.33 ± 0.15	1.26 ± 0.10	0.126	1.34 ± 0.17	1.36 ± 0.18	0.735			
Support time (s)	0.90 ± 0.12	0.85 ± 0.09	0.210	0.91 ± 0.14	0.93 ± 0.16	0.598			
Support phase (%)	67.31 ± 1.93	67.25 ± 2.60	0.930	67.32 ± 1.85	67.97 ± 2.72	0.314			

*p < 0.05; **p < 0.01.

When performing the single task, only the step length in the normal group was significantly longer than that of the cognitive impaired group (*p* < 0.01), which was statistically significant, while there was no significant difference in other results between the two groups. This indicates that the behavior of walking alone has little effect on the cognitively impaired and normal groups, that is, there was no significant difference in gait performance between the two groups when walking alone, according to Table 2.

In the process of DTW-Easy, the step length, step speed, and step frequency of the normal group were greater than those of the cognitively impaired group, with the difference in step length (*p* < 0.05) being statistically significant. And the step time, support time, and support phase of the normal group were all lesser than those of the cognitively impaired group. According to Table 2, this indicates that the counting behavior of the cognitive impaired group during walking has a greater impact on gait performance than normal subjects.

When DTW-Difficult was performed the step length, step speed, and step frequency of the CG consisting of the cognitively healthy people were greater than those of the EG consisting of cognitively impaired older adults people. The difference in step speed (*p* < 0.05) was statistically significant, and the difference of step length (*p* < 0.01) was highly statistically significant. In contrast, the step time, support time, and support phase of the CG were lesser than those of the cognitively impaired group, and the difference in the support phase was statistically significant (*p* < 0.05), indicating that the subtraction calculation behavior in the cognitively impaired group had a stronger effect in gait performance than that of the normal subjects during walking with difficult tasks and because the subtraction calculation behavior was more difficult than the normal DTW-Easy, it also had a stronger effect on gait performance.

#### Analysis of the Results of Indirect Gait Parameters

The measurement of gait performance in older adults is not limited to the examination of direct parameters, but some indirect parameters, such as gait variability and gait dual-task cost, are often examined as important indicators. Among them, the dual-task cost represents the change of brain reserve when subjects need the brain to perform memory retrieval function gait control function. Therefore, the present study analyzed the dual-task cost when performing dual-task walking in normal and cognitively impaired groups.

During DTW-Easy, the dual-task cost in the cognitively impaired group was higher than the normal group, in task 1, and the absolute values of step frequency and step time and support time were significantly different (*p* < 0.01); the cost of dual-tasks in support phase was significantly higher than that in the normal group (*p* < 0.05). According to Table 3, except for the dual-task cost statistics for step, there was no significant difference in speed (*p* > 0.05), but the speed of the cognitive impaired group was still higher.

**Table 3:** The statistics of the dual-task cost during Task 2 and Task 3.

	**Task 2**			**Task 3**		
	**CG**	**EG**	**Significance**	**CG**	**EG**	**Significance**
	**Mean ± SD**	**Mean ± SD**	***p***	**Mean ± SD**	**Mean ± SD**	***p***
Step length	−0.12 ± 7.07	1.22 ± 7.25	0.532	−0.12 ± 7.07	1.22 ± 7.25	0.532
Step speed	−1.20 ± 7.17	−5.32 ± 8.25	0.071	−1.20 ± 7.17	−5.32 ± 8.25	0.071
Step frequency**	−0.82 ± 6.59	−6.41 ± 6.89	0.007	−0.82 ± 6.59	−6.41 ± 6.89	0.007
Step time**	1.26 ± 6.77	7.45 ± 8.67	0.007	1.26 ± 6.77	7.45 ± 8.67	0.007
Support time**	1.26 ± 7.82	8.66 ± 9.98	0.005	1.26 ± 7.82	8.66 ± 9.98	0.005
Support phase*	0.04 ± 1.53	1.08 ± 1.64	0.030	0.04 ± 1.53	1.08 ± 1.64	0.030

*p < 0.05; **p < 0.01.

The results of dual-task costs in the cognitively impaired group during DTW-Difficult were significantly different (*p* < 0.05), because the absolute values of dual-task costs of step length (Table 3), step speed, step frequency, step time, support time, and support phase were greater than those in the normal group. In addition, compared with the results of DTW-Easy, the absolute values of dual-task costs of all gait parameters were greater in DTW-Difficult is greater, indicating that the effects of DTW-Difficult (walking + subtraction behaviors) on subjects’ gait were greater than those caused by DTW-Easy (walking + order counts), which verifies the previous study results, that is, subtraction (sub task of working memory) is more difficult and sensitive.

##### Correlation Analysis Between Dual-Task Cost and the Amount of Change in Connection Strength in the Region of Interest

By analyzing the relationship between the dual-task cost and the change of ROI connection strength in the normal group during the performance of DTW-Easy (Table 4), it is found that the change of connection strength between LPFC-RPFC in the normal group during DTW-Easy is related to step frequency, step time, and support time (*p* < 0.05), as well as the correlation between the dual-task cost of PC-LOC and support phase (*p* < 0.05).

**Table 4:** The analysis of dual-task costs and ROI of CG during Task 2.

**ROI-ROI**	**Dual-Task Costs**	**Correlation**	**Significance**
LPFC-RPFC	Step frequency	−0.33	0.040
	Step time	0.35	0.034
	Support time	0.36	0.026
PC-LOC	Support phase	−0.40	0.012

In the cognitively impaired group (Table 5), during DTW-Easy, it was found that there was a correlation between the change of connection strength between LPFC-PC and the cost of the step task (*p* < 0.01); there was a correlation between the change of connection strength between RPFC-LOC and the dual-task cost in the support phase (*p* < 0.05); the change of connection strength between LTL-PC and LTL-LOC was correlated with the dual-task cost of the step(*p* < 0.05); the change between RTL-PC was correlated with the dual-task cost of step and support phase (*p* < 0.05).

**Table 5:** The analysis of dual-task costs and ROI of EG during Task 2.

**ROI-ROI**	**Dual-task costs**	**Correlation**	**Significance**
LPFC-PC	Step length	0.67	0.005
RPFC-LOC	Support phase	0.61	0.013
LTL-PC	Step length	0.54	0.033
LTL-LOC	Step length	0.51	0.046
RTL-PC	Step speed	0.55	0.029
	Support phase	−0.55	0.027

By analyzing the results during DTW-Difficult for both the normal and cognitively impaired groups (Table 6), it is clear that there was no significant correlation between the amount of change in connection strength between each ROI and the cost of gait dual-task during DTW-Difficult for both the normal group. The cognitively impaired group consists of people with cognitive impairment. Generally, during DTW-Difficult, there is a strong correlation between the change of connection strength between each brain region and the cost of the gait dual-task. Among them, LPFC-RPFC, LPFC-LTL, LPFC-LOC, LPFC-ROC, RPFC-LTL, RPFC-PC, RPFC-ROC, LTL-ROC, and LOC-ROC were predominant.

**Table 6:** The analysis of dual-task costs and ROI of EG during Task 3.

**ROI-ROI**	**Dual-task costs**	**Correlation**	**Significance**
LPFC-RPFC	Step frequency	0.60	0.015
	Step time	−0.67	0.005
	Support time	−0.67	0.004
	Support phase	−0.63	0.010
LPFC-LTL	Support time	−0.50	0.047
	Support phase	−0.52	0.038
LPFC-PC	Step speed	0.55	0.029
LPFC-LOC	Step frequency	0.52	0.041
	Step time	−0.66	0.005
	Support time	−0.67	0.004
	Support phase	−0.58	0.020
LPFC-ROC	Step time	0.64	0.008
	Step frequency	0.73	0.001
	Step time	−0.81	0.000
	Support time	−0.81	0.000
	Support phase	−0.72	0.002
RPFC-LTL	Step speed	0.66	0.006
	Step frequency	0.65	0.006
	Step time	−0.59	0.017
	Support time	−0.58	0.020
	Support phase	−0.60	0.014
RPFC-RTL	Step speed	0.52	0.038
RPFC-PC	Step speed	0.57	0.022
	Step frequency	0.56	0.023
	Step time	−0.52	0.038
	Support time	−0.51	0.044
	Support phase	−0.52	0.040
RPFC-LOC	Step time	−0.50	0.049

### Discussion

In the study of assessing the population with cognitive impairment, a large number of studies focused on the memory, executive function, response-inhibition, and decision-making abilities of the population within the context of traditional cognitive concepts. In recent years, however, an increasing number of studies have focused on motor impairment or gait impairment in cognitively impaired populations. The impact of dual-task walking on gait performance is beginning to receive increasing attention due to the competition mechanisms. In this study, we conducted a dual-task experiment using fNIRS and gait analysis devices on people with cognitive impairment and normal people to investigate the impact of dual-task training modes on the training of cognitively impaired patients.

### Discussion of Direct Results for Gait

The statistical analysis of gait performance between the two groups during task 1 in this study clearly shows that there is usually no significant difference in gait variables (*p* > 0.05), indicating that the effect of walking alone on the gait performance of the normal group and the cognitively impaired group was indistinguishable, and when both groups were trained on a single task, this task had no significant effect on subjects. They were able to devote their full attention to walking behavior. Therefore, there was no difference in the performance of the two groups on this task. This study is also consistent with the results of previous studies (Auvinet et al., 2017).

According to the analysis of the experimental results, the step speed, step length, and step frequency of the cognitive impairment group in DTW-Easy were lower than those of the normal group, while the step time, support time, and support phase were higher than those of the normal group. This indicates that the sub-task of counting the number of dual-task walks had a slightly greater effect on the gait performance of the cognitively impaired group than the healthy individuals. This may be because when performing stepping exercise, the cognitive resources that subjects would have focused on step movement were dispersed in the simple sub-tasks (Taylor et al., 2013; Perrochon et al., 2015).

In DTW-Difficult, subjects were required to perform subtraction while walking, which is a working memory task. Since the subtraction number selected in this study was 7, the number obtained after subtraction was different from the previous one and had no regularity, so it was the most difficult and sensitive for the subjects to set the subtraction number to 7. Therefore, it was observed that the gait performance of both the normal and cognitively impaired groups was affected to varying degrees during DTW-Difficult. The difference between the results of the two groups was generally higher than that of DTW-Easy, and there were significant differences in the results of gait speed, step length, and support phase. This indicates that the subtraction seven sub-task in DTW-Difficult is more difficult and sensitive, and has a more significant effect on the subjects’ gait (Beauchet et al., 2005), which is more obvious in the cognitively impaired group.

### Discussion of the Correlation Results Between the Cost of Gait Dual-Tasking and the Amount of Change in Functional Connectivity

Previous studies have shown that for older adults (Soldan et al., 2015), especially those with cognitive impairment, walking alone is significantly different from walking with another sub-task in terms of brain load. It is interesting to investigate the extent to which the cost of gait dual-tasks correlates with changes in functional connectivity.

In DTW-Easy, the correlation analysis showed that the cost of the gait dual-task did not correlate with the amount of change in functional connectivity between normal and cognitively impaired people, indicating that the counting subtask had no significant effect on the subjects’ gait performance. Both groups were able to overcome the gait disturbance caused by the sub-task, probably due to the lower difficulty and sensitivity of the sub-task. In DTW-Easy, there was still no significant correlation between the cost of the dual-task and the amount of functional connectivity change in the normal population, while the cognitively impaired people showed a high correlation between the cost of the dual-task and the functional connectivity change, indicating that under the influence of more difficult and sensitive sub-tasks, the normal population still had the ability to overcome the interference of sub-tasks on gait. However, the ability of people with cognitive impairment to overcome stronger sub-task interference is relatively insufficient (Bruce-Keller et al., 2012).

### Discussion of the Correlation Results Between Gait Dual-Task Cost and the Amount of Change in ROI Connection

The division of ROI is an important research tool in brain function studies. In this study, the whole brain was divided into seven regions. The connection strength between each ROI represented the cooperation ability between cortical regions corresponding to each ROI. By summarizing the results obtained in this study, it can be seen that there was no significant difference in the results between the normal and cognitively impaired populations during walking alone and walking with counting, while in the process of walking with subtraction, the ROI connection strength of normal people was generally stronger than that of cognitive impairment group. The ROI connection strength of all brain regions in the normal group was generally stronger, while ROI connection strength in the cognitive impairment group was generally reduced. By observation, it can be found that most of the brain regions with increased ROI in the normal population are related to the left prefrontal cortex and the right prefrontal cortex, and the frontal lobe was associated with human cognitive function (Macaulay et al., 2014).

The observation results showed that there was generally no significant correlation between the dual-task cost and the ROI change values of both groups of subjects during walking + counting, indicating that the counting sub-task has little impact on gait performance, and the reserved cognitive resources in the brain of normal and cognitively impaired people were sufficient to overcome the effect of the subtask on gait. In DTW-Difficult, when the subjects performed walking + subtraction, there was still no correlation between the connection strength of each ROI region and the cost of the dual-task in the normal population, while the connection strength of multiple ROI regions in the population with cognitive impairment showed a strong correlation with the cost of the dual-task. This may be because the difficult working memory paradigm had a greater impact on gait and the functional strength of each brain region in the cognitively impaired population (Holtzer and Izzetoglu, 2020). In contrast, no significant effects were observed in the normal population, because the cognitive reserve resources of the normal population were able to overcome the effects of the subtraction sub-task. In addition, the results showed that the connection strength between ROI with a strong correlation between the cognitive impairment population and the dual-task cost mainly depends on the connection strength between the left prefrontal and right prefrontal regions and other regions. Because the frontal lobe is a cortical region that is closely related to human cognitive abilities, so the frontal-temporal synergy is needed to achieve gait control during dual-task control of impaired gait performance, even though the relevant regional connections were more significantly associated with the occurrence of dual-task costs.

The relationship between the strength of correlations and the ability to overcome the effects of sub-tasks has not been clearly determined, but through this study, it can be found that for the cognitively impaired group, sub-tasks with low sensitivity produced lower correlations, while the sub-tasks with higher sensitivity produced higher correlations. This result suggests that by combining sub tasks with high sensitivity, it may be helpful for the diagnosis and identification of the cognitively impaired group.

## Conclusion

Based on fNIRS, this study analyzed the correlation differences between brain functional connectivity and gait variables in people with cognitive impairment during dual-tasks, to provide a reference for the follow-up dual-task studies on the cognitively impaired population. The results showed that there was no significant difference in walking only task between the cognitively impaired group and the cognitively healthy group; however, during the dual-task, compared with the results of task 1, there was a significant difference between the ROI area and the gait of the dual-task. There was a significant difference between task costs, and the cognitively impaired group would be significantly more affected by the sub-task than the normal group when performing DTW-Difficult. This study provides a theoretical basis for clinical use of fNIRS to screen cognitive impairment, contribute to early clinical diagnosis, and can distinguish cognitive impairment group and the normal group by designing more sensitive sub-tasks.

## Limitations

(1) The sample size was 38 cases in the control group and 16 cases in the experimental group. The study results were obtained based on a smaller sample.
(2) The grouping was limited to a simple normal and cognitive impairment group, and the classification of specific subtypes was not further explored.
(3) The interpretation of the results was based on the interpretation of previous gait and fNIRS studies and draws on the study results in the same field, without further validation of the interpretation or speculation of the results.

## Data Availability Statement

The raw data supporting the conclusions of this article will be made available by the corresponding author on reasonable request.

## Ethics Statement

The studies involving human participants were reviewed and approved by The Human Ethics Committee of Jiangbin Hospital. The patients/participants provided their written informed consent to participate in this study.

## Author Contributions

ZW designed the study. DL, ZL, and XH provided updates to the original protocol and interpretation of the study findings. WJ and DW approved the final version of the manuscript. KR recruited participants, collected and analyzed the data, and drafted the manuscript. All authors contributed to the article and approved the submitted version.

## Conflict of Interest

The authors declare that the research was conducted in the absence of any commercial or financial relationships that could be construed as a potential conflict of interest.

## Publisher’s Note

All claims expressed in this article are solely those of the authors and do not necessarily represent those of their affiliated organizations, or those of the publisher, the editors and the reviewers. Any product that may be evaluated in this article, or claim that may be made by its manufacturer, is not guaranteed or endorsed by the publisher.
